# Simultaneous integrated boost on pathologic lymph nodes safely improves clinical outcomes compared to sequential boost in locally advanced cervical cancer: a multicenter retrospective study

**DOI:** 10.3389/fonc.2024.1353813

**Published:** 2024-06-03

**Authors:** Marin Guigo, Mohammed Sali Dauda, Justine Lequesne, Alice Blache, Renata Pereira, Ioana Le Gall, Victor Emmanuel Pernin, Léopold Gaichies, Bénédicte Clarisse, Jean-Michel Grellard, Florence Joly, Emmanuel Meyer, Jacques Balosso

**Affiliations:** ^1^ Department of Radiation Oncology, Centre François Baclesse, Caen, France; ^2^ Clinical Research Department, Centre François Baclesse, Caen, France; ^3^ Department of Radiation Oncology, Centre Hospitalier Universitaire (CHU) Amiens-Picardie, Amiens, France; ^4^ Department of Radiation Oncology, Centre Guillaume Le Conquérant, Le Havre, France; ^5^ Department of Radiation Oncology, Centre de la Baie, Avranches, France; ^6^ Department of Surgery, Centre François Baclesse, Caen, France; ^7^ Institut National de la Santé Et de la Recherche Médicale (INSERM), U1086, ANTICIPE, Cancer and Cognition Platform, Ligue Nationale Contre le Cancer, Medical Oncology Department, Centre François Baclesse, Caen, France; ^8^ Department of Radiation Oncology, Centre Maurice Tubiana, Caen, France

**Keywords:** cervical cancer, nodal boost, pelvic lymph nodes, chemoradiotherapy, intensity-modulated radiation therapy

## Abstract

**Background:**

**Objective:**

This multicenter study aimed to retrospectively evaluate the impact of high boost simultaneous integrated boost (SIB) to pathologic lymph nodes compared to Sequential boost (Seq) in patients with locally advanced cervical cancer (LACC).

**Materials and methods:**

97 patients with pelvic and/or para-aortic (PAo) node-positive LACC treated by definitive chemoradiation were included. Two groups were analyzed: Sequential boost group and simultaneous integrated boost (SIB) group. Endpoints were Distant Recurrence Free Survival (DRFS), Recurrence Free Survival (RFS), Overall Survival (OS), locoregional pelvic and PAo control and toxicities.

**Results:**

3-years DRFS in SIB and Seq groups was 65% and 31% respectively (log-rank *p* < 0.001). 3-years RFS was 58% and 26% respectively (log-rank *p* = 0.009). DRFS prognostic factors in multivariable analysis were SIB, PAo involvement and maximum pelvic node diameter ≥ 2cm. Adenocarcinoma histology and absence of brachytherapy tended to be prognostic factors. SIB provided the best pelvic control at first imaging with 97%. There was no significant difference in terms of toxicities between groups.

**Conclusions:**

Nodal SIB seems to be unavoidable in the treatment of node-positive LACC. It provides the best DRFS, RFS and pelvic control without additional toxicity, with a shortened treatment duration.

## Introduction

Cervical cancer is the 4th cancer in terms of frequency and mortality in the world (1). Despite prevention measures like screening programs and vaccination, many cases are diagnosed at an advanced stage. Pelvic and/or para-aortic (PAo) nodal involvement, found in 40% of locally advanced cervical cancer (LACC) (2), is known to be a major prognostic factor (3). Current standard treatment is concomitant platin-based chemoradiation, using intensity modulated radiotherapy (IMRT) and volumetric modulated arc-therapy (VMAT) to limit toxicities to organs at risk (4), followed by brachytherapy. Excellent local control rates have been reached with the improvement of brachytherapy techniques as image-guided adaptive brachytherapy (5), but pelvic and PAo nodal control remains a major challenge. A high dose boost up to 55 - 60 Gy can be given on pathologic lymph nodes either simultaneously (SIB) or sequentially (Seq), but the latter increases overall treatment duration which is known as a non-negligible prognostic factor in pelvic disease control (6, 7). To our knowledge, no study has compared SIB to Seq in terms of Recurrence Free Survival (RFS), Distant Recurrence Free Survival (DRFS) or Overall Survival (OS). Most studies exploring the impact of nodal boosting were retrospective single-institutional or small prospective studies evaluating SIB and Sequential boost in the same group, against the absence of nodal boost.

In this multi-institutional observational retrospective study, we aimed to determine if SIB to pathologic pelvic and/or PAo lymph nodes provides better clinical outcomes than Seq in LACC, as well as to evaluate toxicities and identify prognostic factors.

## Methods

### Patients’ characteristics

Between January 2014 and December 2021, all patients who were treated in a curative intent by definitive chemoradiation with nodal boost followed or not by brachytherapy for a LACC (stages IIIC1 – IVA FIGO 2018) were included. Data from 5 French hospitals were retrospectively analyzed: François Baclesse Comprehensive Cancer Center, Maurice Tubiana Center, Guillaume Le Conquerant Center, Avranches Private Hospital and Amiens Universitary Hospital. All patients had positive pelvic and/or PAo lymph nodes after undergoing pelvic magnetic resonance imaging (MRI) and positron emission tomography (PET) scan followed by PAo surgical staging if PET was negative at this location. During surgical staging, lymph nodes were dissected up to the inferior mesenteric vein. Maximum lymph node diameter was defined as the shortest axis of the largest lymph node. Patients who had radical hysterectomy or pelvic lymph node dissection were not included. This retrospective study was approved by the institutional review board. It was conducted in compliance with the French Research Standard MR-004 “Research not involving Human participants” (compliance commitment to MR-004 for the Centre François Baclesse number 2214228 v.0, dated from 07/03/2019) and is registered with the French Health Data Hub under the reference F20220712125607. All patients received information and none of them expressed opposition to the use of their data.

### Treatment

Patients underwent whole pelvis external beam radiation therapy (EBRT) using either IMRT, VMAT or 3-Dimension conformational radiation therapy followed by intracavitary brachytherapy, interstitial brachytherapy, or both after clinical and MRI. When brachytherapy was not feasible due to anesthetic or anatomic contraindication, a sequential EBRT boost was given to primary cervical tumor. Patients were attributed two treatment groups according to the management of pathologic lymph nodes: simultaneous integrated boost (SIB) and Sequential boost. SIB was administered with IMRT/VMAT technique and sequential boost with either 3-Dimension conformational radiation therapy or IMRT/VMAT. All patients were receiving concurrent chemotherapy either by weekly cisplatin 40mg/m^2^ or carboplatin AUC2 for patients presenting renal failure or a poor general condition.

### Follow-up and outcomes

Patients all had a follow-up from 3 to 6 months after completion of radiotherapy with clinical examination, MRI and PET or CT scan. Patients were then regularly followed according to each hospital practices, most of the time every 6 months the first 2 years, and then annually. After several years, follow-up could be shared between gynecologist and general practitioner. Acute (during treatment and within 6 months after treatment completion) and late gastro-intestinal and genito-urinary toxicities were reported according to CTCAE V4.0 classification. The primary endpoint was DRFS, defined as the time from the start of radiotherapy to the date of the appearance of a metastasis. Secondary endpoints were RFS defined as the time until recurrence in any location, OS defined as the time until death, pelvic control, PAo control and acute and late toxicities. Pelvic and PAo controls were assessed at the first imaging follow-up. Complete, partial response and stability were considered as favorable for this endpoint.

### Statistical analysis

Patient’s characteristics were described by mean and standard deviation or by median and interquartile range for continuous variables, and by frequencies and proportions for categorical variables. For continuous variables, the comparison of means or medians between the treatment groups was performed with the ANOVA or Kruskal-Wallis test while the proportions of categorical variables were compared with the Pearson’s Chi-squared or the Fisher’s Exact test. Survival probabilities (DRFS, RFS, and OS) were obtained following Kaplan Meier estimation and were compared between the treatment groups using a log-rank test throughout the follow-up period. If there was no event recorded, patients were right-censored on the date they were last seen. Covariates that were significantly associated with survival in univariate analysis and those known to potentially influence survival were integrated into a multivariable Cox proportional hazards model that was fitted to identify prognostic factors through hazard ratios (8). Since this trial was not randomized, treatment allocation depended on certain baseline characteristics (maximum primary tumor diameter, maximum pelvic or PAo lymph node diameter, patient age, tumor (T) and FIGO stages, PAo surgical staging) which were likely to introduce biases and imbalance in the samples. To go further, a propensity-score 2:1 matched method was used to estimate the probability of being assigned a particular treatment conditionally to baseline characteristics by using logistic regression. All statistical analyses were performed using R version 4.1.0.

## Results

### Patients’ characteristics

Patient and disease characteristics are summarized in **Table 1**. A total of 97 patients were included. Median age at the time of diagnosis was 51 years (IQR: 44-64). Squamous cell carcinoma was the most frequent histology (87.9%). Mean primary tumor diameter was 53.7mm, and mean number of positive pelvic nodes was 3. On PET-imaging, 33 (34%) patients had PAo-positive lymph nodes. 10 more patients were classified as PAo-positive after surgical staging that was performed in 42 (43.3%) patients. There was no significant difference between the two groups in rate of PAo- positive patients, number and diameter of pelvic or PAo nodes.

**Table 1:** Patient characteristics.

Factors: Med [IQR]	Seq (n = 21)	SIB (n = 76)	*p*-value
**Age at diagnosis (years)**	51.0 [47.0;59.0]	50.5 [44.0;65.0]	0.94
**ECOG-PS**			0.55
0 - 1	20 (95.2%)	67 (97.1%)	
2	0 (0%)	1 (1.4%)	
3	1 (4.8%)	1 (1.4%)	
**Histology**			1.00
Adenocarcinoma	2 (11.1%)	9 (12.3%)	
Squamous cell carcinoma	16 (88.9%)	64 (87.7%)	
**FIGO (2018) stage**			0.83
IIIC1	11 (52.4%)	37 (48.7%)	
IIIC2	7 (33.3%)	30 (39.5%)	
IVA	3 (14.3%)	9 (11.8%)	
**PAo involvement**	8 (38.1%)	34 (44.7%)	0.59
**PAo surgical staging**			0.65
Yes	10 (47.6%)	32 (42.1%)	
No	11 (52.4%)	44 (57.9%)	
**Tumor diameter (mm)**	55 [50.0;64.0]	50 [40.0;60.8]	0.09
**Max pelvic node diameter (cm)**			0.35
< 2	12 (66.7%)	52 (78.8%)	
≥ 2	6 (33.3%)	14 (21.2%)	
**Number of pelvic nodes**			0.65
< 3	11 (55.0%)	43 (60.6%)	
≥ 3	9 (45.0%)	28 (39.4%)	

Med, Median; IQR, InterQuartile Range; PAo, Para-aortic; ECOG-PS, Eastern Cooperative Oncology Group - Performance Status.

### Treatment

Treatment characteristics are summarized in **Table 2**. 88 (90.7%) patients were treated with VMAT/IMRT technique, and 9 (9.3%) with 3D conformational technique. EBRT doses to the whole pelvis ranged from 43 to 50.4 Gy at 1.8 to 2 Gy per fraction. Total dose on pathologic lymph nodes including boost ranged from 47 to 64.8 Gy (2.2 to 2.4 Gy per fraction) without brachytherapy dose contribution. 76 (78.4%) patients received a SIB and 21 (21.6%) received a sequential boost on pathologic lymph nodes. In SIB group, nodal boost main regimen was 55 Gy at 2.2 Gy per fraction (89%). In Seq group, multiple regimens were used: the main was 10 Gy at 2 Gy per fraction (38%). Mean 2 Gy-equivalent dose (EQD2) to lymph nodes ≥ 2cm was slightly higher than dose to lymph nodes < 2cm in SIB group (56.3 Gy *vs* 55.5 Gy, *p* = 0.036), and in Seq group without significant difference (54.9 Gy *vs* 54.0 Gy, *p* = 0.598). Median overall treatment time was 53 days in SIB group versus 64 days in Seq group. Due to anesthetic or anatomic contraindications, 32 (33%) patients underwent an EBRT boost on primary tumor instead of brachytherapy, without significant difference between groups. Patients were mainly treated with cisplatin concurrently to radiation therapy (90.4%). Mean brachytherapy dose in SIB and Seq groups was 18.6 Gy and 23.7 Gy respectively. In SIB group, mean brachytherapy dose in patients treated with HDR technique was 20 Gy (5 Gy per fraction) and 16.5 Gy for patients treated with PDR technique. In Seq group, mean brachytherapy dose in patients treated with HDR technique was 26 Gy and 23.5 Gy for patients treated with PDR technique.

**Table 2:** Treatment modalities.

Factors	Seq (n = 21)	SIB (n = 76)	*p*-value
**Radiation therapy technique**			<0.0001
3DCRT	9 (42.9%)	0 (0%)	
IMRT/VMAT	12 (57.1%)	76 (100.0%)	
**Total dose on pathologic lymph nodes (Gy, EQD2, α/β = 10)**	Mean: 54.5 Median: 54.3	Mean: 55.4 Median: 55.9	0.224 <0.001
**Primary tumor boost technique**			0.63
EBRT	6 (28.6%)	26 (34.2%)	
Brachytherapy	15 (71.4%)	50 (65.8%)	
**Brachytherapy technique**			<0.001
HDR	1	29	
PDR	14	20	
**Chemotherapy**			0.40
Cisplatin	17 (85.0%)	68 (91.9%)	
Carboplatin	3 (15%)	6 (8.1%)	

SIB, Simultaneous Integrated Boost; Seq, Sequential; 3DCRT, 3-Dimensional Conformational Radiation Therapy; EBRT, External Beam Radiation Therapy; EQD2, 2Gy-equivalent dose; HDR, High Dose Rate; PDR, Pulsed Dose Rate; IMRT, Imaging Modulated Radiation Therapy; VMAT, Volumetric Modulated Arc-Therapy.

### Outcomes

**Figure 1:**
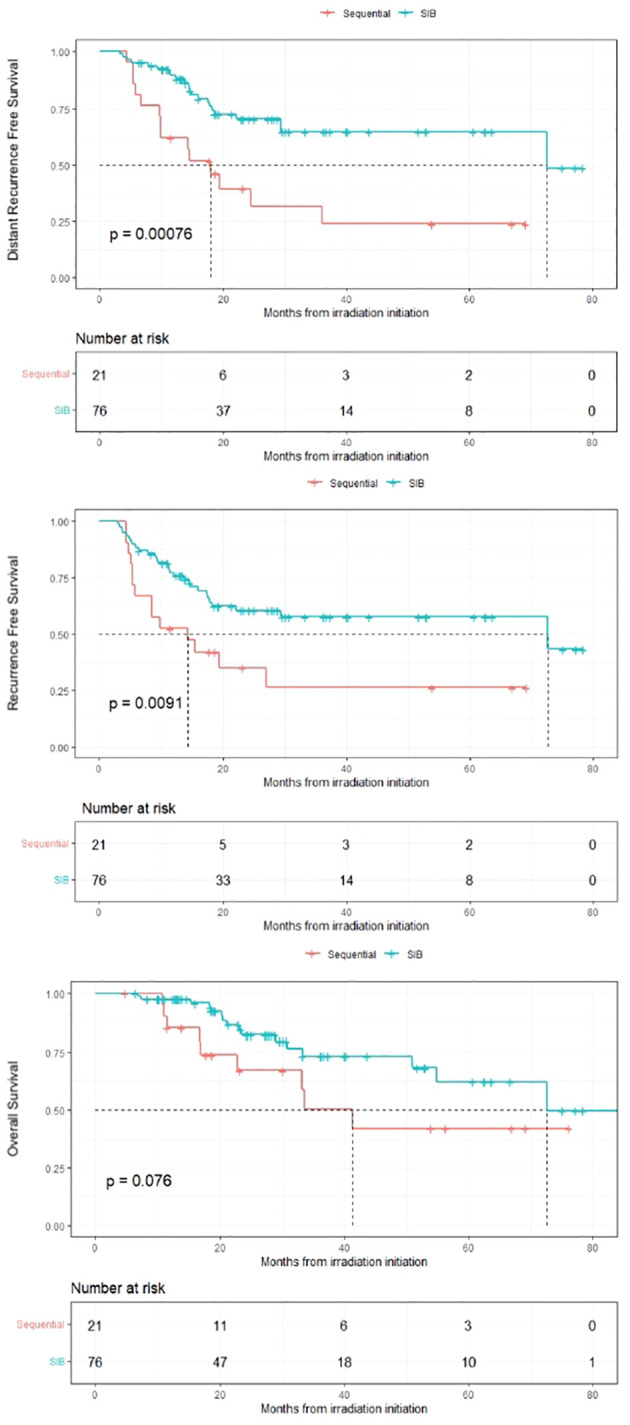
Kaplan-Meier survival curves for patients with locally advanced cervical cancer receiving pelvic irradiation with simultaneous or sequential boost on pathologic nodes SIB = Simultaneous Integrated Boost.

Survival results are depicted in **Figure 1**. Prognostic factors according to multivariable analyses are depicted in **Table 3** and according to propensity matched multivariable analyses in Additional File 1. Median follow-up in Seq and SIB groups was 54 and 28 months respectively. Propensity-score 2:1 matched analysis allowed to match 36 SIB patients with 18 Seq patients, for a total sample of 54 patients.

**Table 3:** Multivariable analyses of factors influencing DRFS, RFS and OS.

Factors	DRFS		RFS		OS	
	HR (95% CI)	*p*-value	HR (95% CI)	*p*-value	HR (95% CI)	*p*-value
PAo nodes involvement						
Yes	4 (1.51-11.1)	**0.005**	1.82 (0.79-4.35)	0.2	1.59 (0.5-5)	0.4
No	—		—		—	
Histology						
Adenocarcinoma	2.56 (0.92-7.14)	0.07	4.17 (1.75-10)	**0.001**	4 (1.18-14.29)	**0.027**
Squamous cell carcinoma	—		—		—	
Max pelvic node diameter (cm)						
< 2	—		—		—	
≥ 2	2.70 (0.96-7.57)	0.059	1.61 (0.63-4.11)	0.3	1.14 (0.23-5.58)	0.9
**Tumor diameter**	1.02 (1-1.06)	0.11	1.02 (0.99-1.05)	0.14	1.04 (1 -1.07)	0.055
Number of pelvic nodes						
< 3	—		—		—	
≥ 3	1.1 (0.44-2.72)	0.8	0.95 (0.41-2.19)	> 0.9	1.19 (0.39-3.63)	0.8
Concurrent chemotherapy						
Carboplatin	—		—		—	
Cisplatin	0.38 (0.12-1.25)	0.11	0.3 (0.10-0.88)	**0.028**	0.30 (0.08-1.07)	0.063
Primary tumor boost technique						
EBRT	2.44 (0.94-6.31)	0.066	1.40 (0.61-3.23)	0.4	2.38 (0.75-7.59)	0.14
Brachytherapy	—		—		—	
Nodal boost						
SIB	0.23 (0.10-0.56)	**0.001**	0.4 (0.18-0.89)	**0.025**	0.24 (0.07-0.75)	**0.01**
Sequential	—		—		—	

HR, Hazard Ratio; CI, Confidence Interval; DRFS, Disease Recurrence Free Survival; RFS, Recurrence Free Survival; OS, Overall Survival; PAo, Para-aortic; SIB, Simultaneous Integrated Boost; EBRT, External Beam Radiation Therapy. Significative differences are put in bold.

### Distant recurrence free survival

3-years DRFS in SIB and Seq groups was 65% and 31% respectively (log-rank *p* < 0.001). On multivariable analyses, SIB was independently protective compared to Seq boost (HR: 0.23; 95% CI: 0.10-0.56, *p* = 0.001), as well as on propensity matched multivariable analyses (*p* = 0.009). Other DRFS independent prognostic factors in multivariable analyses were PAo node involvement (HR: 4; 95% CI: 1.51-11.1, *p* = 0.005) and maximum pelvic node diameter ≥ 2cm (HR: 2.7; 95% CI 0.96-7.57, *p* = 0.059), close to significance. Adenocarcinoma histology compared to squamous cell carcinoma (HR: 2.56; 95% CI: 0.92-7.14, *p* = 0.072) and absence of brachytherapy (HR: 2.44; 95% CI 0.94-6.31, *p* = 0.067) tended to be independent prognostic factors.

### Recurrence free survival

3-years RFS was 58% in SIB group and 26% in Seq group (log-rank *p* = 0.009). SIB was protective in multivariable analyses (HR: 0.40; 95% CI: 0.18-0.89, *p* = 0.025) as in propensity matched multivariable analyses, although it was not significant (HR: 0.5; 95% CI: 0.20-1.23, *p* = 0.13). Adenocarcinoma histology and concomitant carboplatin instead of cisplatin were independent prognostic factors in both multivariable and weighted multivariable analyses.

### Overall survival

There was a trend towards a difference in 3-years OS between SIB and Seq groups (73% and 50% respectively (log-rank *p* = 0.076)). SIB was protective for this endpoint in multivariable analyses (HR: 0.24; 95% CI: 0.07-0.75, *p* = 0.015) and main prognostic factor was adenocarcinoma histology.

### Locoregional control

Pelvic nodal control at first imaging follow-up in SIB and Seq groups was 97% and 86% respectively (*p* = 0.066). PAo nodal control was 96% in SIB and 81% in Seq group (*p* = 0.038).

### Toxicities

Acute and late GI and GU toxicities are depicted in **Table 4**. There was no significant difference in terms of grade ≥ 3 toxicities between groups.

**Table 4:** Acute and tardive gastrointestinal and genito-urinary toxicities according to CTCAE v4.0 classification.

Toxicity		Seq (n = 21)	SIB (n = 76)	*p*-value
Acute GI toxicity	0-2	18 (90.0%)	62 (81.6%)	0.5101
	≥ 3	2 (10.0%)	14 (18.4%)	
	MD	1	0	
Acute GU toxicity	0-2	20 (100.0%)	74 (97.4%)	> 0.9
	≥ 3	0 (0%)	2 (2.6%)	
	MD	1	0	
Late GI toxicity	0-2	21 (100.0%)	75 (100%)	_
	MD	0	1	
Late GU toxicity	0-2	21 (100.0%)	75 (100%)	_
	MD	0	1	

GI, gastro-intestinal; GU, genito-urinary; MD, missing data.

## Discussion

The purpose of this study was to assess the benefit and safety of SIB on pathologic lymph nodes compared to sequential boost in a context of locally advanced cervical cancer. To our knowledge, this study is the first retrospective multicenter study to compare the outcomes of these two boost modalities on nodal and distant disease control.

In our study, SIB provided significantly higher 3-years DRFS and RFS than Seq boost. OS and pelvic nodal control at first evaluation were also higher, close to significance. This difference can be explained by a longer total duration of treatment with Seq boost, which is known to be an important predictive factor of treatment response (9, 10). SIB achieved high rates of locoregional control, which is consistent with Tiwari et al. prospective study showing regional control rates of 93% in nodal boost group versus 80% in the absence of boost. In their study, no significant difference were found in terms of Disease Free Survival (DFS) and OS, but 60% of nodal group patients underwent a sequential boost and only 40% a SIB (11). This choice could explain why most studies didn’t showed significant benefits of nodal dose escalation in terms of distant or overall survival.

Furthermore, SIB did not significantly add digestive or urinary toxicity compared to Seq boost, which is consistent with Feng et al. study showing that SIB provide better sparing of organs at risk than sequential nodal boost (12). In Dang et al. study, SIB was well tolerated with only 5.5% patients who suffered from tardive grade ≥ 3 GI toxicity, all characterized by rectal symptoms (13). Even compared with the absence of boost, Jayatilakebanda et al. showed no additional grade ≥ 3 GI or GU toxicity with the use of SIB (14).

The use of a propensity-score weighting method was a strength of our study. It allowed to reduce the risk of selection bias by re-equilibrating groups conditionally to baseline characteristics. Main prognostic factors identified in both multivariable and weighted regression models were adenocarcinoma histology, primary tumor diameter, maximum pelvic lymph node diameter ≥ 2cm and PAo lymph node involvement. Gogineni et al. showed an inferior nodal control in patients with ≥ 2cm lymph nodes at 12 months (77% *vs* 100%, *p* = 0.002). In this particular population, dose escalation > 50.4 Gy on ≥ 2cm lymph nodes provided a better lymph node control (100% *vs* 60%, *p* = 0.02) (15). In our study, 22 (28.9%) patients in SIB group and 10 (47.6%) patients in Seq group relapsed in the radiation field (*p* = 0.19). Regarding all failures, 37 (42%) patients treated with IMRT and 6 (67%) patients treated with 3D conformational radiotherapy relapsed (p = 0.179). In patients who experienced a failure, mean EQD2 to adenopathies was 54.9 Gy versus 55.5 Gy for patients who did not relapse (*p* = 0.257). Even if it was not significant, number and largest diameter of pathologic lymph nodes were higher in Seq group, which could have influenced clinical outcomes. In Bacorro and al study, 108 patients with 254 nodes underwent EBRT and brachytherapy for a locally advanced cervical cancer: at a median follow-up of 33.5 months, 38% of patients relapsed. No patient treated with nodal SIB experienced a failure. Nodal control probability was influenced mainly by nodal volume with a threshold of 3 cm^3^, and nodal dose > 57.5 Gy EQD2 (*p* = 0.039) (16). These studies suggest that boost dose should be escalated according to the size of pathologic lymph nodes.

As for OS, DRFS can be influenced by the treatment of local or locoregional recurrences. However, according to studies that analyzed recurrence patterns, distant metastatic failure as a first recurrence is quite common despite excellent pelvic locoregional control (11, 17). From our point of view, DRFS was therefore a legitimate primary endpoint to analyze.

One-third of patients did not receive brachytherapy due to accessibility, anesthetic or anatomic reasons which, on one hand, limits comparability to other studies, but on the other hand makes the study applicable to centers that do not perform brachytherapy and instead pursue primary tumor treatment with EBRT. Brachytherapy dose contribution to pathologic lymph nodes was not taken into account in the calculation of the total received dose, due to complexity of retrospectively retrieving these data in medical records. However, according to the literature, this dose is estimated to be between 2 and 6 Gy EQD2 to the internal and external iliac lymph nodes, and less than 2 Gy EQD2 to the common iliac and para-aortic region (18).

As a weakness of the study, median follow-up was relatively short, which may have resulted in a lack of power to show a significant difference in OS or to identify other prognostic factors. As SIB technique was implemented recently in current practice, median follow-up was shorter in SIB group (28 months) than Seq group. For the purpose of extrapolation, we choose to analyze survival at 3 years, meaning that SIB group survival after 28 months should be carefully interpreted.

Nevertheless, despite the decreasing number of LACC in the population, the multicenter nature of the study allowed for a relatively sufficient number of patients with node involvement compared to other studies. In addition, it enabled good extrapolation of the results to general population, knowing variations in practices between hospitals. All patients had PET-imaging at diagnosis and a majority of patients were treated with IMRT, which allows to extrapolate our data to current practices. Soon expected results of prospective multicenter interventional EMBRACE II study will provide survival data on a large cohort of patients, using the most advanced treatment techniques (19). Recently updated ESGO/ESTRO/ESP guidelines recommend to give a SIB to macroscopic pathologic nodes up to 60 Gy EQD2 including brachytherapy dose contribution, with a IIIB (“prospective cohort studies, strong or moderate evidence for efficacy but with a limited clinical benefit, generally recommended”) level of evidence (20). If IMRT/VMAT technique is available, sequential nodal boost should not be realized.

## Conclusion

Considering the benefit provided by SIB in terms of locoregional control, DRFS and RFS with the absence of additional toxicity and a shortened treatment duration, it appears legitimate to offer this treatment modality to patients suffering from LACC with pathologic pelvic and/or PAo lymph nodes. In order to highlight a benefit of SIB in OS, interventional prospective studies with larger cohorts need to be conducted.

## Data availability statement

The raw data supporting the conclusions of this article will be made available by the authors, without undue reservation.

## Ethics statement

Ethical approval was not required for the study involving humans in accordance with the local legislation and institutional requirements. Written informed consent to participate in this study was not required from the participants or the participants’ legal guardians/next of kin in accordance with the national legislation and the institutional requirements.

## Author contributions

MG: Writing – review & editing, Writing – original draft, Visualization, Methodology, Investigation, Data curation, Conceptualization. MD: Writing – review & editing, Visualization, Methodology, Investigation, Formal analysis, Data curation. JL: Writing – review & editing, Visualization, Methodology, Investigation, Formal analysis, Data curation. AB: Writing – review & editing, Investigation. RP: Writing – review & editing. IL: Writing – review & editing. V-EP: Writing – review & editing. LG: Writing – review & editing. BC: Writing – review & editing, Methodology. J-MG: Writing – review & editing, Methodology. FJ: Writing – review & editing, Visualization, Supervision, Conceptualization. EM: Writing – review & editing, Visualization, Supervision, Methodology, Investigation, Conceptualization. JB: Writing – review & editing, Visualization, Supervision, Methodology, Investigation, Conceptualization.
