# Supplementary material for: Simultaneous integrated boost on pathologic lymph nodes safely improves clinical outcomes compared to sequential boost in locally advanced cervical cancer: a multicenter retrospective study
Source: Front Oncol. 2024 Jun 3;14:1353813. doi: 10.3389/fonc.2024.1353813 (PMC11180790; doi:10.3389/fonc.2024.1353813)
Supplement: Supplementary file 1 [file Table_1.docx]

| **Factors** | **DRFS** | | **RFS** | | **OS** | |
| --- | --- | --- | --- | --- | --- | --- |
|  | **HR (95% CI)** | ***p*-value** | **HR (95% CI)** | ***p*-value** | **HR (95% CI)** | ***p*-value** |
| **PAo nodes involvement** |  | |  | |  | |
| Yes | 4.35 (1.14-16.67) | **0.031** | 3.45 (1.10-11.11) | **0.035** | 1.61 (0.31-8.33) | 0.6 |
| No | — | | — | | — | |
| **Histology** |  | |  | |  | |
| Adenocarcinoma | 2.63 (0.47-14.29) | 0.3 | 7.14 (1.56-33.33) | **0.011** | inf |  |
| Squamous cell carcinoma | — | | — | | — | |
| **Max pelvic node diameter (cm)** |  | |  | |  | |
| < 2 | — | | — | | — | |
| > 2 | 4.02 (1.17-13.8) | **0.03** | 2.68 (0.9-8.04) | 0.078 | 1.91 (0.34-10.6) | 0.5 |
| **Tumor diameter** | 1.04 (0.99-1.08) | 0.12 | 1.06 (1.01-1.1) | **0.007** | 1.05 (0.99-1.12) | 0.10 |
| **Number of pelvic nodes** |  | |  | |  | |
| < 3 | — | | — | | — | |
| > 3 | 0.77 (0.23-2.57) | 0.7 | 0.58 (0.2-1.64) | 0.3 | 0.69 (0.17-2.77) | 0.6 |
| **Concurrent chemotherapy** |  | |  | |  | |
| Carboplatin | — | | — | | — | |
| Cisplatin | 0.47 (0.11-1.99) | 0.3 | 0.23 (0.06-0.89) | **0.033** | 0.28 (0.07-1.18) | 0.082 |
| **Primitive tumor boost technique** |  | |  | |  | |
| EBRT | 1.35 (0.40-4.59) | 0.6 | 0.56 (0.19-1.69) | 0.3 | 0.68 (0.11-4.33) | 0.7 |
| Brachytherapy | — | | — | | — | |
| **Nodal boost** |  | |  | |  | |
| SIB | 0.26 (0.09-0.72) | **0.009** | 0.5 (0.20-1.23) | 0.13 | 0.31 (0.08-1.24) | 0.1 |
| Sequential | — | | — | | — | |

**Additional File 1:** Multivariable analyses of factors influencing DRFS, RFS and OS according to propensity-score matched samples

HR = Hazard Ratio, CI = Confidence Interval, DRFS = Disease Recurrence Free Survival, RFS = Recurrence Free Survival, OS = Overall Survival, PAo = Para-aortic, SIB = Simultaneous Integrated Boost, EBRT = External Beam Radiation Therapy, inf = infinite
